# Schizophrenia patients with a metabolically abnormal obese phenotype have milder negative symptoms

**DOI:** 10.1186/s12888-020-02809-4

**Published:** 2020-08-18

**Authors:** Juan Wang, Yulong Zhang, Zhiwei Liu, Yating Yang, Yi Zhong, Xiaoshuai Ning, Yelei Zhang, Tongtong Zhao, Lei Xia, Feng Geng, Rui Tao, Mei Fan, Zhenhua Ren, Huanzhong Liu

**Affiliations:** 1 Department of Psychiatry, Chaohu Hospital of Anhui Medical University, 64 Chaohu North Road, Hefei, 238000 China; 2 Anhui Psychiatric Center, Anhui Medical University, Hefei, China; 3 Department of psychiatry, Fuyang Third People’s Hospital, Anhui, China; 4 Department of Psychiatry, Hefei Fourth People’s Hospital, Hefei, China; 5 Department of Anatomy, Anhui Medical University, 81 Meishan Road, Hefei, 230000 China

**Keywords:** Schizophrenia, Negative symptoms, Metabolically abnormal obese, Metabolically healthy normal-weight, Metabolic syndrome

## Abstract

**Background:**

Schizophrenia patients with a metabolically abnormal obese (MAO) phenotype have been shown poor cardiovascular outcomes, but the characteristics of their current psychiatric symptoms have not been characterized. This study mainly explored the psychiatric symptoms of schizophrenia patients with the MAO phenotype.

**Methods:**

A total of 329 patients with schizophrenia and 175 sex- and age-matched people without schizophrenia from Anhui Province in China were enrolled. The Positive and Negative Syndrome Scale (PANSS) was used to evaluate the mental symptoms of the schizophrenia patients. The MAO phenotype was defined as meeting 1–4 metabolic syndrome criteria (excluding waist circumference) and having a body mass index (BMI) ≥ 28 kg/m^2^. And, metabolically healthy normal-weight (MHNW) phenotype was defined as meeting 0 criteria for metabolic syndrome and 18.5 ≤ BMI < 24 kg/m^2^.

**Results:**

Overall, 15.8% of the schizophrenia patients and 9.1% of the control group were consistent with the MAO phenotype, and the prevalence of MAO in the schizophrenia group was higher than that in the control group. Among the patients with schizophrenia, the MAO group had lower negative factor, cognitive factor and total PANSS scores than the MHNW group. However, when confounding factors were controlled, only the negative factor remained lower significantly.

**Conclusion:**

We found that schizophrenia patients with the MAO phenotype had reduced negative symptoms, which may indicate an internal mechanism linking metabolic disorders and negative symptoms.

**Trial registration:**

This study was registered in the China Clinical Trial Registration Center (No. chiCTR 1,800,017,044).

## Background

Schizophrenia is a disease associated with high disability and high mortality [1–3]. Existing studies have shown that the life expectancy in patients with schizophrenia is 10 to 20 years shorter than that in people without schizophrenia [4]. One of the leading causes of premature death in patients with schizophrenia is cardiovascular disease [5–7]. Among the risk factors for cardiovascular disease, obesity is prominent and very common [8, 9].

Researchers have recently divided obesity into two phenotypes: metabolically abnormal obese (MAO) and metabolically healthy but obese (MHO) [10–13]. In recent years, studies have found that MAO people have the highest risk of cardiovascular disease and the worst cardiovascular prognosis, followed by MHO people; metabolically healthy normal-weight (MHWN) individuals have the lowest risk and the best cardiovascular prognosis [14–16].

Patients with schizophrenia have a high incidence of metabolic side effects and obesity due to the use of second-generation antipsychotics, such as clozapine, olanzapine, and quetiapine [17–19]. Additionally, because of the specific lifestyle characteristics of people with schizophrenia, such as poor diet [20], sedentary lifestyle [21] and lack of exercise [22], the incidence of obesity is increased in this population. Therefore, it is necessary to closely attend to the specific group--schizophrenia patients with MAO phenotype.

However, there is no report on the prevalence of the MAO phenotype in people with schizophrenia. Additionally, it is not clear whether the prevalence of the MAO phenotype is higher in schizophrenia patients than in the general population. In addition, while studies have identified poor cardiovascular outcomes in MAO individuals, the current mental state of MAO individuals with schizophrenia has not been reported; is it also poor? This is also the purpose of our research. Therefore, our study mainly explored the prevalence of MAO in patients with schizophrenia and compared it with that of the control group. Furthermore, it was deemed important to explore the current mental symptoms of MAO individuals with schizophrenia.

## Methods

### Subjects

All patients with schizophrenia were recruited from the inpatient wards of three hospitals in Anhui Province in China between May to December 2018. On the basis of the inclusion criteria and exclusion criteria, 443 inpatients were consecutively enrolled in the study. A total of 333 inpatients completed the study. Of the 110 patients who did not complete the study, 9 were unwilling to provide blood samples, 72 were unable to cooperate with the interview, 13 refused to participate, 9 were discharged, and 7 were unknown. The data of an additional 4 patients were incomplete and were excluded. Therefore, 329 patients with schizophrenia were ultimately included in this study. All schizophrenia patients were diagnosed by clinicians using the International Classification of Diseases-10th edition (ICD-10). The age range of the patients was 18–75 years and the illness duration was more than 5 years (all were chronic patients [23]). After understanding the purpose and method of the study, 175 sex- and age-matched volunteer were recruited as the control group for this study at the health check-up centre in hospital which is an integrated place to provide health examination and assess the health status for residents. The control group has no history of schizophrenia in the past, and in this study, the control group was not found to have mental abnormalities by psychiatrists. The control groups were aged 18 to 75 years old. The schizophrenia patients and the control participants were excluded when the following conditions were present: severe neurological disease; intellectual disability; substance dependence excluding alcohol and tobacco; and pregnancy or lactation. All subjects volunteered to participate in the study; they or their guardians signed an informed consent form.

### Definition of the MAO phenotype

Currently, in international studies, MAO is defined in individuals who meet two requirements: metabolic abnormality and obesity [8]. There are two methods for defining metabolic abnormalities: the diagnostic criteria for metabolic syndrome (MS) and insulin resistance/sensitivity cut-off points [8]. To unify the research and facilitate comparisons, the Circulation Research Compendium on Obesity, Diabetes and Cardiovascular Disease recommends using the MS definition for metabolic abnormalities [8]. Regarding the criteria for obesity, we adopted the recommendation of WHO experts for Asian populations [24], that is, body mass index (BMI) ≥ 28 kg/m^2^ was classified as obesity [25].

In summary, the inclusion criteria for subjects with the MAO phenotype were as follows: (1) meeting 1–4 diagnostic criteria for MS (excluding waist circumference) and (2) BMI ≥ 28 kg/m^2^. The unified criteria for MS in 2009 [26] were as follows: (1) triglyceride level ≥ 1.69 mmol/L or hypolipidemic drug use; (2) high-density lipoprotein cholesterol (HDL-C) < 1.04 mmol/L for the male sex, HDL-C < 1.29 mmol/L for the female sex, or therapeutic drug use; (3) systolic blood pressure (SBP) ≥ 130 mmHg and/or diastolic blood pressure (DBP) ≥ 85 mmHg or antihypertensive drug use; and (4) fasting blood glucose ≥5.6 mmol/l or hypoglycaemic drug use. In addition, we selected MHNW patients as the control group according to the following criteria: 0 MS criteria and 18.5 ≤ BMI < 24 kg/m^2^.

### Sociological and clinical characteristics

Data on sex, age, marital status, education level, current smoking status and food intake were collected for all subjects. Current smoking behaviour was defined as smoking more than 1 cigarette per day more than 5 days per week in the last 2 weeks. Since the main food source for the people of Anhui Province in China is rice, we have only roughly estimated the consumption of rice. According to the Chinese version of the Food Frequency Questionnaire, the Chinese standard small bowl of rice was 100 g [27] . Based on the subjects’ recollection of their daily eating habits, we classified a usual food intake of less than 100 g of rice per meal as grade I, and more than or equal to 100 g of rice per meal as grade II. Height and weight were measured after the subjects took off their shoes and while they were wearing light clothes. The calculation of BMI was calculated using weight (kg) / height (m)^2^ [28]. We collected the schizophrenia patients’ disease information, including the age at onset, the total course of the disease, the history of accompanying chronic somatic diseases such as hypertension or diabetes, and the types and dose of antipsychotic medications currently in use. All currently used drug doses were converted to chlorpromazine equivalents [29].

### Biochemical detection

Fasting blood samples were taken between 6 and 8 am and were sent to the clinical laboratory for examination. Fasting blood glucose was measured by the oxidase method (Meikang Biotechnology Co., Ltd., Zhejiang, China). Triglyceride was measured by the GPO-PAP method (Beijing Lidan Biochemical Co., Ltd., Beijing, China). Cholesterol was measured by the CHOD-POD method (Beijing Lidan Biochemical Co., Ltd., Beijing, China). HDL-C and LDL-C were measured by the terminal method (Neusoft Whitman Biotechnology (Nanjing) Co., Ltd., Jiangsu, China). Fasting plasma insulin and plasma C-peptide levels were both measured by electrochemiluminescence (Roche Diagnostics GmbH, Mannheim, Germany). Glucagon was tested by radioimmunoassay (Beijing North Biotechnology Research Institute Co.,Ltd., Beijing, China). Insulin resistance (IR) refers to low insulin sensitivity and abnormal glucose metabolism. IR was quantitatively calculated by homeostasis model assessment (HOMA), and the formula was HOMA-IR = (fasting blood glucose * fasting plasma insulin) / 22.5 [30]. We defined HOMA-IR values greater than 2.5 as insulin resistance [31].

### Scale evaluation

We used the 30-item Positive and Negative Syndrome Scale (PANSS) to evaluate the severity of psychiatric symptoms in patients with schizophrenia [32]. Four trained interviewers administered the PANSS, and the internal consistency coefficient was greater than 0.8. Each item of the PANSS was scored from 1(asymptomatic) to 7 (extremely symptomatic), and the higher the total scores were, the more serious the mental symptoms. We used the five-factor model of the PANSS to evaluate the mental symptoms from multiple dimensions. The five-factor model [33] includes the following: (1) positive factor (P1, P3, P5, G9), (2) negative factor (N1, N2, N3, N4, N6, G7), (3) cognition factor (P2, N5, G11), (4) excited factor (P4, P7, G8, G14), and (5) depressed factor (G2, G3, G6). The six items comprising the PANSS negative factor are blunted affect, emotional withdrawal, poor rapport, passive/apathetic social withdrawal, lack of spontaneity, and motor retardation.

### Data analysis

First, we compared the characteristics between the patients with schizophrenia and the control group. For continuous variables, the independent samples t-test and Mann-Whitney U test were used as appropriate. For categorical variables, the Chi-square test was used. Second, only in patients with schizophrenia, the clinical characteristics of the MAO and MHNW groups of patients were compared. An independent samples t-test, Mann-Whitney U test and Chi-square test were also used as appropriate. Third, to control for all possible confounding factors and further compare the psychiatric symptoms in the MAO and MHNW groups, we performed a binary logistic regression with the “Enter” method. The groups (MAO and MHNW) were the dependent variables (MHNW group as the reference group), and the other variables, including sociodemographic and clinical data, the types and dose of antipsychotics, and PANSS factor scores, were simultaneously included as covariates. Forth, in patients with schizophrenia, the Spearman correlation (most variables not normally distributed) was used to explore the correlation between MS components and diabetes related variables (fasting blood glucose, insulin, C-peptide, glucagon, HOMA-IR). All statistical analyses were performed with SPSS 23.0, and a two-tailed *P* value < 0.05 indicated significance.

## Results

### Comparison between the patients with schizophrenia and the controls

A total of 329 patients with chronic schizophrenia and 175 sex- and age-matched people without schizophrenia were recruited for this study. The frequencies of MAO and MHNW phenotypes in patients with schizophrenia were 15.8 and 9.4%, respectively, and the corresponding values for the control subjects were 9.1 and 14.9%, respectively. The MAO phenotype of patients with schizophrenia was significantly higher than that of the control group (*p* = 0.037). In addition, among the obese subjects, the proportions of the MAO phenotype were 96.3 and 84.2% in the patients and the control groups, respectively.

The demographic characteristics and clinical data of patients and controls were shown in Table 1. The marriage prevalence, food intake, education level and HDL-C level were lower in the patients than in the control group. The BMI, triglyceride level, LDL-C level, and plasma C-peptide level were higher in the schizophrenia group than in the control group.

**Table 1:** The characteristics and clinical data of patients with schizophrenia and the control participants

	Schizophrenia patients		Control participants			
	*N* = 329		*N* = 175			
	*N*	*%*	*N*	*%*	*χ*^*2*^	*P*
Female	134	40.7	81	46.3	1.44	0.230
Married	95	30.1	139	82.7	126.05	< 0.001
Current smokers	97	29.7	44	25.1	3.90	0.272
Insulin resistance	103	31.9	43	24.7	2.81	0.094
MAO phenotype	52	15.8	16	9.1	4.35	0.037
MHNW phenotype	31	9.4	26	14.9	3.36	0.067
Food intake						
I	98	30.3	24	14.6	14.29	< 0.001
II	225	69.7	140	85.4		
	*Mean*	*SD*	*Mean*	*SD*	*Z/t*	*P*
Age (years)	45.23	11.72	46.08	13.33	−0.82	0.410
Education (years)	8.11	3.63	9.46	3.79	−3.69	< 0.001
BMI (kg/m^2^)	24.12	3.84	23.45	3.16	1.96	0.049
Glucose (mmol/l)	5.36	1.36	5.33	1.08	−0.37	0.711
Cholesterol (mmol/l)	4.76	1.41	4.58	0.89	−0.89	0.373
Triglycerides (mmol/l)	2.24	1.51	1.63	1.06	−6.08	< 0.001
HDL _C (mmol/l)	1.05	0.27	1.16	0.27	−4.75	< 0.001
LDL_C (mmol/l)	2.40	0.63	2.23	0.53	2.97	0.003
C-peptide (ng/ml)	2.75	1.14	2.54	1.32	−2.79	0.005
Insulin (mU/L)	9.48	8.08	8.69	8.29	−1.81	0.070
HOMA-IR	2.37	2.97	2.20	3.00	−1.57	0.116

*MAO phenotype* Metabolically abnormal obese phenotype, *MHNW phenotype* Metabolically healthy normal-weight phenotype, *BMI* Body mass index, *HDL_C* High-density lipoprotein cholesterol, *LDL_C* Low-density lipoprotein cholesterol, *insulin resistance* HOMA-IR ≥ 2.5, *HOMA-IR* Homeostasis model assessment of insulin resistance

### Comparison between the MAO and MHNW groups of patients with schizophrenia

As shown in Table 2, we classified 52 patients into the MAO group and 31 patients into the MHNW group. The MAO group had more females, higher education levels and lower chlorpromazine equivalents than the MHNW group. The MAO group had worse metabolic status than the MHNW group, as indicated by higher C-peptide levels, insulin levels, glucagon levels, HOMA-IR index values, and insulin resistance rates. Regarding psychiatric symptoms, the patients in the MAO group had lower negative factor, cognitive factor and PANSS total scores than those in the MHNW group.

**Table 2:** Comparison between the MAO group and the MHNW group for patients with schizophrenia

	Total (*n* = 83)		MAO (*n* = 52)		MHNW (*n* = 31)			
	*N*	*%*	*N*	*%*	*N*	*%*	*χ*^*2*^	*P*
Female	34	41.0	28	53.8	6	19.4	9.55	0.002
Married	21	25.3	15	28.8	6	19.4	2.36	0.500
Current smokers	24	28.9	13	25.0	11	35.5	2.06	0.356
Insulin resistance	32	40.0	29	56.9	3	10.3	16.67	< 0.001
Food intake								
I	24	29.6	13	26.0	11	35.5	0.825	0.364
II	57	70.4	37	74.0	20	64.5		
Antipsychotics								
Clozapine	36	43.4	24	46.2	12	38.7	0.438	0.508
Olanzapine	16	19.3	10	19.2	6	19.4	0.01	0.989
Risperidone	28	33.7	16	30.8	12	38.7	0.55	0.459
Aripiprazole	15	18.1	8	15.4	7	22.6	0.68	0.410
	*Mean*	*SD*	*Mean*	*SD*	*Mean*	*SD*	*Z/t*	*P*
Age (years)	43.36	12.44	43.40	12.40	43.29	12.73	0.04	0.968
Education (years)	7.63	4.01	8.42	4.04	6.29	3.65	−2.80	0.005
Age of onset (years)	26.33	8.12	26.79	9.73	25.55	8.31	0.671	0.504
Duration of illness (years)	16.94	10.18	16.42	9.73	17.81	10.99	−0.39	0.695
hospitalization days	109.47	103.18	120.62	125.02	92.73	54.50	−0.731	0.465
Chlorpromazine equivalents (mg/day)	457.38	277.47	404.47	241.49	546.13	313.54	−2.31	0.024
BMI (kg/m^2^)	26.86	4.72	30.21	1.74	21.23	1.82	22.35	< 0.001
Glucose (mmol/l)	5.33	1.35	5.57	1.63	4.93	0.44	−1.78	0.075
Cholesterol (mmol/l)	4.81	1.17	5.11	1.24	4.32	0.86	3.12	0.003
Triglycerides (mmol/l)	2.37	1.77	3.13	1.87	1.13	0.35	−7.18	< 0.001
HDL _C (mmol/l)	1.07	0.25	0.95	0.19	1.28	0.19	−7.59	< 0.001
LDL_C (mmol/l)	2.45	0.58	2.56	0.55	2.28	0.60	2.13	0.036
Glucagon (pg/ml)	28.29	20.26	34.75	20.86	17.46	13.73	4.11	< 0.001
C-peptide (ng/ml)	3.09	1.37	3.66	1.36	2.08	0.60	5.95	< 0.001
Insulin (mU/L)	12.16	12.55	15.74	14.33	5.74	3.07	−5.68	< 0.001
HOMA-IR	3.24	5.20	4.36	6.24	1.26	0.70	−5.73	< 0.001
PANSS								
Positive factor	9.95	4.58	9.96	4.54	9.94	4.71	0.025	0.980
Negative factor	18.39	7.42	15.94	6.89	22.48	6.49	−4.28	< 0.001
Cognitive factor	8.98	2.96	8.44	3.22	9.87	2.25	−2.17	0.033
Depressive factor	6.88	2.87	7.00	3.02	6.68	2.65	0.49	0.624
Excited factor	7.71	3.59	7.67	3.78	7.77	3.30	−0.13	0.899
PANSS total	78.65	23.84	74.50	25.46	85.61	19.30	−2.10	0.039

*MAO* Metabolically abnormal obese, *MHNW* Metabolically healthy normal-weight, *BMI* Body mass index, *HDL_C* High-density lipoprotein cholesterol, *LDL_C* Low-density lipoprotein cholesterol, *insulin resistance* HOMA-IR ≥ 2.5, *HOMA-IR* Homeostasis model assessment of insulin resistance, *PANSS* Positive and negative syndrome scale

To eliminate possible confounding factors and compare psychiatric symptoms, we performed binary logistic regression analyses with the “Enter” method. The covariables included sex, age, education level, age of onset, duration of disease, hospitalization days, food intake, marital status, smoking status, types of antipsychotic medicines, chlorpromazine equivalents, and the PANSS factor scores. The significant difference in negative factor between the two groups persisted (*p* = 0.003, OR = 1.29, 95% CI = 1.09–1.52), but the difference in cognitive factor (*p* = 0.112, OR = 1.24, 95% CI = 0.95–1.62) and PANSS total scores (*p* = 0.066, OR = 1.03, 95% CI = 0.99–1.06) disappeared.

### Correlation between MS components and diabetes related variables in patients with schizophrenia

As shown in Table 3, the Spearman correlation showed that diabetes related variables were associated with almost all metabolic indicators. Insulin, C-peptide and HOAM-IR were positively correlated with BMI, glucose, cholesterol, triglyceride, LDL-C, SBP and DBP, and negatively correlated with HDL-C. Blood glucose was positively correlated with BMI, cholesterol, triglyceride and LDL-C. Glucagon was positively correlated with BMI, triglyceride and DBP, and negatively correlated with HDL-C.

**Table 3:** The correlation between the MS components and diabetes related indicators in patients with schizophrenia (*N* = 329)

	Glucose	Insulin	C-peptide	Glucagon	HOMA-IR
BMI	0.121^*^	0.477^***^	0.482^***^	0.295^***^	0.475^***^
Glucose	–	0.159^**^	0.131^*^	0.047	0.412^***^
Cholesterol	0.120^*^	0.183^***^	0.150^**^	0.073	0.206^***^
Triglycerides	0.169^**^	0.395^***^	0.360^***^	0.248^***^	0.403^***^
HDL _C	−0.095	−0.162^**^	−0.221^***^	− 0.190^***^	−0.174^**^
LDL_C	0.160^**^	0.171^**^	0.136^*^	0.081	0.208^***^
SBP	0.024	0.143^**^	0.163^**^	0.101	0.151^**^
DBP	−0.011	0.176^***^	0.225^***^	0.172^**^	0.179^***^

Spearman correlation was used for statistical analysis. The data in the table was correlation coefficients and the asterisk indexed significant. *BMI* Body mass index, *HDL_C* High-density lipoprotein cholesterol, *LDL_C* Low-density lipoprotein cholesterol, *SBP* Systolic blood pressure, *DBP* Diastolic blood pressure, *HOMA-IR* Homeostasis model assessment of insulin resistance

^*^P < 0.05

^**^P < 0.01

^***^P < 0.001

## Discussion

In this study, we found that the prevalence of the MAO phenotype in patients with chronic schizophrenia and individuals without schizophrenia were 15.8 and 9.1% respectively. To the best of our knowledge, this is the first report of the prevalence of the MAO phenotype in people with schizophrenia. Most previous studies have reported in the general population. In a study in Ireland, based on three different definitions of metabolic abnormalities (NCPE/ATP III MS criteria, HOMA-IR criteria, and Wildman criteria), the rate of the MAO phenotype in the general population was 19.8–23.8% (BMI ≥ 30 kg/m^2^) [34]. A survey in Fujian Province in China reported that the prevalence of MAO in the general population was 34.6% (BMI ≥ 24 kg/m^2^, metabolic abnormalities ≥1 NCPE/ATP III MS criterion or HOMA-IR > 2.69) [35]. The differences in these studies are mainly due to different diagnostic criteria, race, population etc.

The prevalence of the MAO phenotype in patients with schizophrenia was higher than in the general population, which was also the first report of this finding. The use of second generation antipsychotics resulted in adverse reaction, mainly including metabolic abnormalities and obesity. Antipsychotics can act on adipose tissue, destroy adipose tissue cells, and create intracellular imbalance, resulting in metabolic disorders in the body and causing IR [17]. Existing studies have also found that antipsychotic drug receptors such as H1 [36], 5-HT 2A, 5-HT 2C, D2 [37], D3, and M3 [38] are associated with energy metabolism, feeding and weight and contribute to weight gain and obesity [39]. In addition, less exercise [22] and poor diet [20] in patients with schizophrenia can also lead to weight gain.

We found that most diabetes-related variables (for example HOMA-IR) were positively correlated with MS components except HDL_C, and negatively correlated with HDL_C. This suggested that worse metabolic levels were closely related to higher risk of diabetes. The result was similar to some previous studies [40]. Some internal connections about them had been reported. The main pathological mechanism of type 2 diabetes was the decrease of insulin receptor sensitivity, that is, IR, resulting in relative lack of insulin [41]. Chronic, low-level inflammation of adipose tissue macrophages in obesity was an important contributor to IR [42]. During IR, the circulatory function of free fatty acids was impaired, resulting in an increase in triglyceride and LDL_C, a decrease in HDL_C, and lipid accumulation in liver and muscle tissue [43]. And insulin has a direct vasodilating effect, and the average vasodilatory response was in the range 15–30% under the physiological dose, but this effect was weakened in patients with IR or diabetes [44, 45]. In summary, the metabolic level was closely related to insulin function, and the worse the metabolic level, the higher the risk of diabetes.

In this study, we found a phenomenon contrary to conventional wisdom. Schizophrenia patients with the MAO phenotype had milder negative symptoms compared to patients with the MHNW phenotype and the difference remained after controlling for confounding variables. Mezquida et al. reported a negative correlation between BMI and negative symptoms [46], which was somewhat similar to the findings of this study. Virawudh et al. reported that IR was strongly positively associated with negative symptoms after controlling for BMI [47]. IR is the central link of MS and is essentially a “synonym” of abnormal metabolism. In our study, when metabolic abnormalities and obesity were combined, the negative symptoms were alleviated.

Schizophrenia patients with the MAO phenotype had mild negative symptoms, and this indicated that the worse the metabolism, the milder the negative symptoms. The underlying pathophysiologic mechanisms were not clear. Regarding this, we propose three possible hypotheses. First, we guessed it may be related to the use of antipsychotics. Antipsychotics can relieve negative symptoms, and also can lead to metabolic side effects [48]. In our MAO sample, the metabolic status of patients was worse, but the negative symptoms were milder. We believe that these patients with worse metabolism may respond better to antipsychotics, and have better therapeutic effects, resulting in milder negative symptoms. In addition, the types of antipsychotics also have a great impact on negative symptoms and metabolism. For example, olanzapine is more likely to lead to metabolic side effects than aripiprazole, and is more effective in treating negative symptoms [48]. So, prescriptions for antipsychotics may have a potential impact on the results. Psychiatrists may prefer antipsychotics that are effective against psychiatric symptoms, even if they have metabolic side effects [49]. Second, S.F. Chen et al. reported that after controlling for drug use, negative symptoms were negatively correlated with triglyceride levels and positively correlated with HDL-C levels [50]. The authors suggested that negative symptoms may be related to a peculiar lipid pathological mechanism [50]. Third, we think that the reward system may be involved. In patients with schizophrenia, the reward system is damaged due to the dysfunction in dopamine transmission [51]. Patients with more serious negative symptoms had lower reward responses and reward adaptation, which led to a decrease in the ability and accuracy to obtain rewards [52]. Obesity has been associated with the food reward [53]. Some studies have found that a stronger reward response was associated with a higher BMI [54]. Therefore, there might be a reward mechanism. In MAO patients with lower negative symptoms, their reward responsiveness may be less impaired, with relatively more eating rewards remaining, which led to obesity.

There may be some unknown physiological mechanisms in regard to the presence of mild negative symptoms in MAO patients. It is necessary to continue exploring the internal relationships between metabolic status and negative symptoms in patients with schizophrenia. It is expected that new methods that can alleviate negative symptoms without worsening metabolism will be found.

There were few shortcomings in this research. First, this was a cross-sectional study, which prevents conclusions of causality. Further cohort studies are needed to determine the internal relationships between abnormal metabolic obesity and negative symptoms. Second, the sample size was relatively small, and our results need to be replicated and expanded in a larger population. However, to avoid sample selection bias, our study was conducted at three different locations. Third, our schizophrenia sample included only inpatients, control group was a non-hospitalized population, and the different lifestyles of the two groups may have biased the results of study. Fourth, all the patients were chronic schizophrenia patients, with a long duration of illness. The drug previously taken by patients extended too far into the past to be verified. The antipsychotic drugs and doses noted in this study refer only to currently administered drugs.

## Conclusion

In this study, we reported, for the first time, the prevalence of the MAO phenotype in patients with schizophrenia and compared it with the prevalence in the general population. We also found that the mental symptoms of the MAO phenotype in patients with schizophrenia were not more serious but were actually less severe, and this was observed mainly with the negative symptoms. We speculate that there may be some related pathophysiological mechanisms between metabolic abnormalities and negative symptoms. Further cohort studies and basic research are needed to explore these mechanisms. We hope to find a way to alleviate the negative symptoms without worsening metabolism.

## Data Availability

The datasets used and/or analysed during the current study are available from the corresponding author on reasonable request.

## Abbreviations

MAO: Metabolically abnormal obese
MHO: Metabolically healthy but obese
MHNW: Metabolically healthy normal-weight
MS: Metabolic syndrome
BMI: Body mass index
HDL-C: High-density lipoprotein cholesterol
LDL-C: Low-density lipoprotein cholesterol
SBP: Systolic blood pressure
DBP: Diastolic blood pressure
IR: Insulin resistance
HOMA-IR: Homeostasis model assessment-insulin resistance
PANSS: Positive and Negative Syndrome Scale
